# Cycling performance decrement is greater in hypobaric versus normobaric hypoxia

**DOI:** 10.1186/2046-7648-3-8

**Published:** 2014-04-28

**Authors:** Beth A Beidleman, Charles S Fulco, Janet E Staab, Sean P Andrew, Stephen R Muza

**Affiliations:** 1 Thermal and Mountain Medicine Division, United States Army Research Institute of Environmental Medicine, Kansas St., Bldg 42, Natick, MA 01760, USA

**Keywords:** Endurance performance, Altitude, Hypobaric hypoxia, Resting ventilation, Time trial performance

## Abstract

**Background:**

The purpose of this study was to determine whether cycling time trial (TT) performance differs between hypobaric hypoxia (HH) and normobaric hypoxia (NH) at the same ambient PO_2_ (93 mmHg, 4,300-m altitude equivalent).

**Methods:**

Two groups of healthy fit men were matched on physical performance and demographic characteristics and completed a 720-kJ time trial on a cycle ergometer at sea level (SL) and following approximately 2 h of resting exposure to either HH (*n* = 6, 20 ± 2 years, 75.2 ± 11.8 kg, mean ± SD) or NH (*n* = 6, 21 ± 3 years, 77.4 ± 8.8 kg). Volunteers were free to manually increase or decrease the work rate on the cycle ergometer. Heart rate (HR), arterial oxygen saturation (SaO_2_), and rating of perceived exertion (RPE) were collected every 5 min during the TT, and the mean was calculated.

**Results:**

Both groups exhibited similar TT performance (min) at SL (73.9 ± 7.6 vs. 73.2 ± 8.2), but TT performance was longer (*P* < 0.05) in HH (121.0 ± 12.1) compared to NH (99.5 ± 18.1). The percent decrement in TT performance from SL to HH (65.1 ± 23.6%) was greater (*P* < 0.05) than that from SL to NH (35.5 ± 13.7%). The mean exercise SaO_2_, HR, and RPE during the TT were not different in HH compared to NH.

**Conclusion:**

Cycling time trial performance is impaired to a greater degree in HH versus NH at the same ambient PO_2_ equivalent to 4,300 m despite similar cardiorespiratory responses.

## Background

Due to increasing interest in using hypoxic training to induce improvements in endurance performance in athletes, mountaineers, and military personnel, the use of intermittent exposures to either hypobaric hypoxia (HH) or normobaric hypoxia (NH) as an adjunct or in addition to regular sea-level training has generated a plethora or research [1-7]. Whereas NH can be recreated anywhere using a mask, bag, or commercially available room, HH requires multi-million dollar hypobaric chambers or natural altitude conditions. The ease of use of NH compared to HH has generated questions on whether repeated exposures using this emerging technology is beneficial for enhancing endurance performance. The results from most of these studies are controversial and depend on whether the endurance tests are conducted at sea level or altitude; the severity, length, and dose of hypoxic exposure; and the type of study design [8-12].

No studies to date have determined whether endurance performance differs during *acute* exposures to HH and NH. If NH does not induce the same degree of hypoxic stimulus as HH, its usefulness as a training aid for endurance performance improvements at sea level or altitude may be limited. One meta-analysis suggested that live-high, train-low protocols conducted in HH were more effective for enhancing sea-level endurance performance than live-high, train-low protocols conducted in NH [10]. However, this meta-analysis provided no insight on whether endurance performance is the same or different in HH and NH. More recently, a review suggested that repeated exposures to HH or NH conditions resulted in distinctly different endurance performance outcomes during subsequent exposure to terrestrial altitude [8]. Collectively, these reviews [8,10] indicate that similar training under HH or NH conditions do not provide similar endurance performance outcomes during subsequent exposure to sea level or terrestrial altitude conditions. Whether these performance differences are related to differences in physiological processes occurring during acute exposures to HH and NH is not well understood.

A recent series of articles presented diverging stances on whether the physiologic responses to HH and NH are similar or different [13-15]. Some studies reported 20% to 30% increases in resting ventilation in NH compared to HH [16-19], while other studies reported no differences between the two environments at altitudes equivalent to 4,000 m and above [20-22]. In most studies that reported increased ventilation in NH, arterial oxygen saturation (SaO_2_), the end product of both pulmonary ventilation and gas exchange, was similar between the two environments [17-19]. To date, most studies have reported no differences in the cardiac and hematologic responses to HH and NH [18,19,22-24]. As such, it is currently unclear and hotly debated as to whether exposure to HH and NH elicits different physiologic responses.

The purpose of this study was to determine in two groups of men, matched on physical performance and demographic characteristics, whether cycling time trial (TT) performance differs during an acute exposure to HH and NH at the same ambient PO_2_ (93 mmHg, 4,300-m altitude equivalent). We hypothesized a greater decrement in cycle TT performance in HH compared to NH due to increased ventilation and improved oxygen delivery in NH.

## Methods

### Volunteer test subjects

Two groups of healthy fit men were exposed to either HH (*n* = 6) or NH (*n* = 6). These two groups were matched on sea-level (SL) cycle TT performance (mean ± SD; 73.9 ± 7.6 min vs. 73.2 ± 8.2 min), SL peak oxygen uptake (VO_2peak_) (47.5 ± 4.3 ml/kg/min vs. 49.5 ± 5.0 ml/kg/min), age (20 ± 2 years vs. 21 ± 3 years), height (178 ± 7 cm vs. 177 ± 5 cm), and weight (75.2 ± 11.8 kg vs. 77.4 ± 8.8 kg). Each of the 12 volunteers was a lifelong, low-altitude resident and had no exposure to altitudes greater than 1,000 m for at least 6 months immediately preceding the study. All received medical examinations, and none had any condition warranting exclusion from the study, including pulmonary hypertension, sickle cell trait, or family history of migraine. All tested within normal ranges for pulmonary function and had normal hemoglobin [Hb] and serum ferritin levels. All volunteers performed regular sea-level aerobic training (1–2 h · week^-1^) before and during the study. Each gave written and verbal acknowledgment of their informed consent and was made aware of their right to withdraw without prejudice at any time. The study was approved by the Institutional Review Board of the US Army Research Institute of Environmental Medicine in Natick, MA, USA. Investigators adhered to the policies for protection of human subjects as prescribed in Army Regulation 70-25, and the research was conducted in adherence with the provisions of 45 CFR Part 46.

### Study design

**Figure 1:**
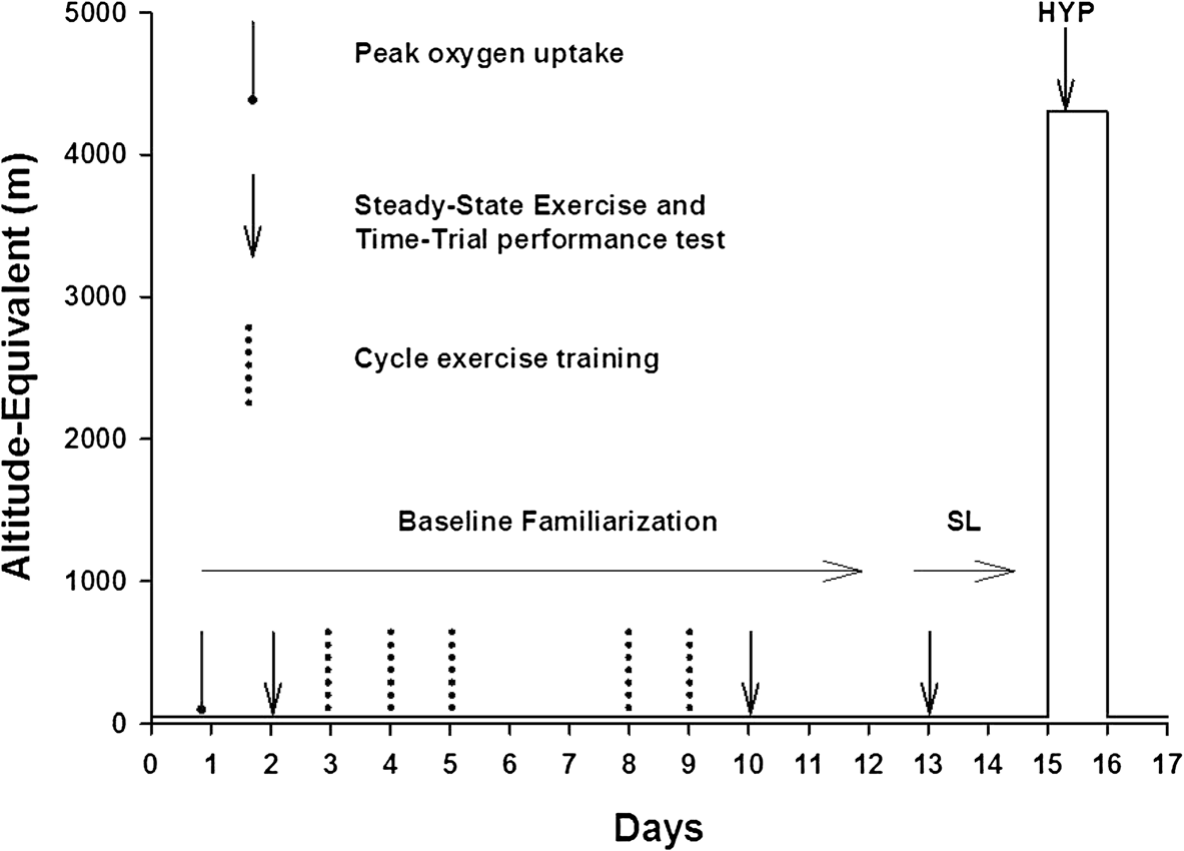
**Study design indicating timing of baseline, SL, and HYP measurements in either HH or NH.** One preliminary peak oxygen uptake, two preliminary cycle endurance tests consisting of two steady-state exercise bouts followed by a cycle time trial test, and five cycle exercise training sessions occurred during baseline testing. Prior to each cycle endurance test in SL and HYP, resting ventilatory, cardiovascular, and hematologic responses were measured.

The study design is shown in Figure 1. This study used an unblinded two-factor (test condition and group) experimental design. The test conditions were defined as SL and hypoxia (HYP). The groups were defined as HH and NH. Preliminary SL baseline measurements included the following: (1) a VO_2peak_ test, (2) a cycle endurance test, (3) five 45-min cycle training sessions conducted at 55% VO_2peak_, and (4) a second cycle endurance test. These preliminary measurements were made to stabilize performance improvements due to familiarization with the cycle ergometer and/or training effects. The VO_2peak_ was not measured in HYP but estimated by decrementing the SL VO_2peak_ by 26% which is an established group decrement at 4,300 m [25]. Data collection included the following measurements made in both SL and HYP in the following order: (1) body weight in t-shirt and shorts, (2) resting ventilation, (3) resting blood sample, and (4) cycle endurance test, and (5) post-exercise blood sample. The cycle endurance test consisted of five distinct segments: (1) a 5-min warm-up at 50 W, (2) steady-state exercise consisting of 20 min at 45% altitude-specific VO_2peak_ and 20 min at 65% altitude-specific VO_2peak_, (3) a post-exercise blood sample, (4) a 5-min break to allow bathroom use and stretching, and (5) a self-paced 720-kJ cycle TT done as fast as possible. The total exposure time to HYP was approximately 4 h with the resting measures occurring in the first 2 h and cycle endurance test occurring in the second 2 h.

### Peak oxygen uptake

VO_2peak_ was measured during incremental, progressive cycling exercise to exhaustion at SL only. Volunteers pedaled for 2 min at 50, 100, and 150 W. Following 2 min at 150 W, the workload was increased in 30-W increments for 2-min stages until O_2_ uptake failed to increase or they stopped the test despite strong encouragement. During the test, oxygen consumption was measured continuously using a metabolic cart (True Max 2400, ParvoMedics, Salt Lake City, UT, USA), heart rate (HR) was monitored continuously using a heart rate watch (UNIQ CIC, Computer Instruments Co., Hempstead, NY, USA), arterial oxygen saturation (SaO_2_) was measured continuously by finger pulse oximetry (Model 8600, Nonin Medical Inc., Plymouth, MN, USA), and a rating of perceived exertion (RPE) was determined every 2 min using the 6 to 20 Borg scale [26].

### Resting ventilation and cardiovascular assessment

Resting ventilation was measured in the morning prior to the cycle endurance test both at SL and in HYP. Volunteers sat in a semi-recumbent position and breathed through a low-resistance breathing circuit connected to a breath-by-breath open-circuit metabolic system (Vmax 229, Viasys Healthcare, Yorba Linda, CA, USA) calibrated with certified gases and volume standard. Each volunteer's resting minute ventilation (VE), oxygen consumption (VO_2_), carbon dioxide production (VCO_2_), and end-tidal oxygen and carbon dioxide partial pressure (PETO_2_ and PETCO_2_) were measured. Simultaneously, SaO_2_ and HR were measured by pulse oximetry. Ventilation data was collected for at least 10 min, and the mean over the last 5–8 min of the session was calculated. Resting systolic blood pressure (SBP) and diastolic blood pressure (DBP) were measured after completing ventilation measures, and mean arterial pressure (MAP) was calculated as 0.333 (SBP - DBP) + DBP.

### Hematologic assessment

Immediately following the resting ventilation measurement, a resting venous blood sample was obtained from the forearm for the measurement of hemoglobin ([Hb]) and hematocrit (Hct) both at SL and in HYP. Following the steady-state portion of the cycle endurance test, another blood sample was obtained for measurement of [Hb] and Hct. The samples were analyzed immediately in duplicate using the i-STAT portable clinical analyzer (Abbott Diagnostics, Abbott Park, IL, USA). Percent change in plasma volume (PV) from SL to HYP and from pre- to post-exercise in both SL and HYP was calculated according to the Dill equation [27].

### Steady-state exercise testing

Cycle endurance tests were performed on an electromagnetically braked cycle ergometer (Excalibur, Lode BV, Groningen, Netherlands). At rest and during the two steady-state work bouts, HR, SaO_2_, and RPE were collected as previously described and respiratory gas measurements (i.e., VE, VO_2_, VCO_2_) were made using open-circuit spirometry (Vmax 229, Viasys Healthcare, Yorba Linda, CA, USA). The metabolic cart was calibrated with certified gases and volume standard and used in the mixing chamber mode for exercise testing. The respiratory exchange ratio (RER) was calculated from VO_2_ and VCO_2_ measurements. The ventilatory equivalents for O_2_ (VE · VO_2_^-1^) and CO_2_ (VE · VCO_2_^-1^) were calculated from each subject's VE, VO_2_, and VCO_2_ data. Cardiac output (CO) was also measured at rest and during the two steady-state work rates using a validated non-invasive continuous finger blood pressure measurement system (Finometer, Finapres Measurement Systems, Arnhem, Netherlands) [28-30].

### Time trial performance

After the steady-state exercise, each volunteer completed a 720-kJ TT on a cycle ergometer. Respiratory gas measurements were not made during the TT in order to eliminate the impact of breathing through a mouthpiece on an all-out endurance performance effort. A shorter duration to complete the fixed 720-kJ time trial from one testing condition to another was considered an improvement in performance and vice versa. Due to the extensive familiarization period, volunteers were well acquainted with their capabilities on the cycle ergometer and were free to choose the initial wattage at the start of the TT. During the TT, volunteers were visually aware of the current wattage on the cycle ergometer at all times and free to manually increase or decrease the work rate by 5-W increments. There were no restrictions on the number or direction of 5-W changes. Volunteers were continually informed of the distance completed but not the time elapsed during the TT. This type of TT performance test has been shown to have a high repeatability and low coefficient of variation [31]. The HR, SaO_2_, and RPE by methods previously described were collected every 5 min during the TT, and the mean value was calculated.

### Environmental conditions

All testing was performed at SL in Natick, MA, USA in a hypobaric chamber or hypoxia room (Colorado Altitude Training, Inc., Boulder, CO, USA) maintained at a temperature and relative humidity of 21 ± 2°C and 45 ± 5%, respectively. The hypoxia room was maintained at an ambient PO_2_ of 93 mmHg by reducing the fractional inspired concentration of O_2_, while the hypobaric chamber was maintained at an ambient PO_2_ of 93 mmHg by decompression to 446 mmHg over a 15-min period. The inspired PO_2_ in the hypobaric chamber was 83.4 mmHg, while the inspired PO_2_ in the hypoxia room was 87.4 mmHg. The ambient PO_2_ was maintained in the normobaric hypoxia room with a combination of automatic feedback loops using the True Max gas analyzers as well as manual adjustments in flow to add room air if ambient PO_2_ levels dropped from desired levels. The ambient PO_2_ fluctuated very little (±0.1 mmHg) due to constant monitoring of the room. The SL testing was performed at ambient barometric pressure (approximately 760 mmHg), and all HYP exposures were conducted at an altitude equivalent of 4,300 m. The mean ambient PCO_2_ values in the hypoxia room (0.9 ± 0.2 mmHg) and hypobaric chamber (0.7 ± 0.2 mmHg) were similar.

### Statistical analyses

A two-way repeated measures ANOVA was used to analyze differences between the repeated measures test condition factor (SL and HYP) and independent group factor (HH and NH) for all resting ventilatory and cardiovascular assessments as well as cycle TT performance. Three-way ANOVAs, with repeated measures on the additional factor of exercise duration, were used for physiological measurements during the two steady-state work rates. Pearson product-moment correlations were utilized to determine significant associations between variables of interest. Significant main effects and interactions were analyzed using Tukey's least significant difference test. Effect size was calculated as a 15 ± 8 min difference in cycle TT performance between groups. This effect size was based on the standard deviation from previous TT data collected in our laboratory [32] as well as detecting a large threshold effect [33]. Six volunteers per group were required to achieve a power of 0.8. Statistical significance was set at *P* < 0.05. All data are presented as means ± SD.

## Results

The cycle TT improved (i.e., faster time) from the first to second preliminary test at SL in both the HH group (96.7 ± 5.0 min vs. 79.4 ± 4.9 min) and NH group (93.7 ± 12.6 min vs. 79.3 ± 9.8 min). However, the cycle TT did not change from the second preliminary test to the first cycle TT at SL for either the HH (73.9 ± 7.6 min) or NH (73.2 ± 8.2 min) group. Thus, any learning or training effects due to unfamiliarity with the cycle ergometer were minimized prior to definitive data collection.

### Resting ventilation, cardiovascular, and hematologic data

Resting ventilatory and cardiovascular measurements are presented in Table 1. None of the resting measurements were significantly different between the two groups prior to beginning steady-state exercise at SL or in HYP. Resting SaO_2_ and PETO_2_ were lower in HYP compared to SL values, but other physiologic measures did not change from SL. The percent change in resting PV from SL to HH (4.8 ± 7.5%) or SL to NH (6.1 ± 8.9%) was also not different between groups or from zero.

**Table 1:** Resting ventilatory, cardiovascular, and hematologic responses in hypobaric hypoxia (HH) and normobaric hypoxia (NH)

**Group**	**Condition**	**VE (BTPS) (l · min**^**-1**^**)**	**PETO**_**2 **_**(mmHg)**	**PETCO**_**2 **_**(mmHg)**	**SaO**_**2 **_**(%)**	**HR (bpm)**	**MAP (mmHg)**	**Hb (mg/dl)**	**Hct (%)**
HH (*n* = 6)	SL	11.1 ± 2.2	105.8 ± 5.8	39.4 ± 3.3	97.2 ± 1.4	68.0 ± 9.8	85.5 ± 4.1	15.9 ± 0.7	46.7 ± 1.9
	HYP	13.0 ± 3.1	47.2 ± 2.4*	40.1 ± 4.3	79.6 ± 4.2*	71.8 ± 8.3	86.8 ± 6.5	15.5 ± 0.7	45.6 ± 2.2
NH (*n* = 6)	SL	10.8 ± 1.3	102.8 ± 2.3	39.0 ± 1.9	98.0 ± 0.9	67.7 ± 3.0	86.5 ± 6.2	15.7 ± 1.1	46.1 ± 3.3
	HYP	11.1 ± 1.6	46.6 ± 2.8*	36.8 ± 1.6	78.5 ± 3.4*	70.7 ± 2.6	84.2 ± 2.9	15.2 ± 0.6	44.7 ± 1.9

Mean ± SD; *P < 0.05 from SL; ^†^*P* < 0.05 between groups. *SL* sea level, *HYP* hypoxia, *VE* minute ventilation, *PETO*_*2*_ partial pressure of end-tidal oxygen, *PETCO*_*2*_ partial pressure of end-tidal carbon dioxide, *SaO*_*2*_ arterial oxygen saturation, *HR* heart rate, *MAP* mean arterial pressure, *[Hb]* hemoglobin concentration, *Hct* hematocrit.

### Steady-state exercise data

Ventilatory, cardiovascular, and perceptual responses measured during the two steady-state exercise bouts on the cycle ergometer are presented in Table 2. Given the 26% estimated decrement in VO_2peak_ from SL to HYP, the work rates (W) at 45% and 65% of altitude-specific VO_2peak_ were decreased (*P* < 0.05) in HYP compared to SL in both groups. Between groups, there were no differences in the 45% or 65% altitude-specific VO_2peak_ work rates at SL or HYP. These results attest to adequate matching of volunteers and were thus ‘by design’.

**Table 2:** Ventilatory, cardiovascular, and perceptual responses in a HH group and NH group at SL and in HYP

**Group**	**Condition**	**% peak**	**Work rate (W)**	**VE(BTPS) (l · min**^**-1**^**)**	**VO**_**2 **_**(l · min**^**-1**^**)**	**VE · VO**_**2**_^**-1**^	**VE · VCO**_**2**_^**-1**^	**SaO**_**2 **_**(%)**	**HR (bpm)**	**CO (l · min**^**-1**^**)**	**MAP (mmHg)**	**RPE**
HH (*n* = 6)	SL	45	110 ± 8	43 ± 7	1.55 ± 0.10	27.7 ± 5.0	33.3 ± 5.0	98 ± 1	126 ± 11	14.9 ± 2.5	105 ± 9	8 ± 2
		65	157 ± 14	67 ± 9	2.13 ± 0.13	31.2 ± 4.3	37.2 ± 4.3	97 ± 1	159 ± 13	17.5 ± 2.1	108 ± 11	11 ± 3
	HYP	45	73 ± 8*	39 ± 11	1.11 ± 0.12*	35.2 ± 3.4*	35.7 ± 3.4	74 ± 5*	126 ± 13	13.9 ± 1.3	109 ± 14	8 ± 2
		65	110 ± 8*	61 ± 5	1.53 ± 0.12*	40.2 ± 2.5*	39.8 ± 2.5*	75 ± 3*	149 ± 11	16.1 ± 2.1	115 ± 24	11 ± 3
NH (*n* = 6)	SL	45	116 ± 23	44 ± 9	1.57 ± 0.26	29.4 ± 3.0	29.6 ± 3.0	98 ± 1	130 ± 19	13.1 ± 2.5	115 ± 9	9 ± 2
		65	162 ± 24	67 ± 17	2.03 ± 0.39	33.2 ± 4.7	33.1 ± 4.7	97 ± 1	156 ± 17	14.7 ± 2.6	113 ± 12	13 ± 2
	HYP	45	68 ± 24*	37 ± 7	1.18 ± 0.25*	30.6 ± 3.3	33.6 ± 3.3*	74 ± 6*	121 ± 15	12.8 ± 1.7	109 ± 7	10 ± 2
		65	116 ± 24*	61 ± 13	1.66 ± 0.36*	36.5 ± 3.0*	38.4 ± 3.0*	75 ± 4*	148 ± 13	15.6 ± 2.7	117 ± 11	12 ± 3

Mean ± SD; *P < 0.05 from SL at same relative work rate within groups; ^†^*P* < 0.05 between groups at same relative work rate. *SL* sea level, *HYP* hypoxia, *VE, BTPS* minute ventilation, *VO*_*2*_ oxygen uptake, *VE · VO*_*2*_^*-1*^ ventilatory equivalent for oxygen, *VE · VCO*_*2*_^*-1*^ ventilatory equivalent for carbon dioxide, *SaO*_*2*_ arterial oxygen saturation, *HR* heart rate, *CO* cardiac output, *MAP* mean arterial pressure, *RPE* rating of perceived exertion; 45% of altitude-specific VO_2peak_ (45%), 65% of altitude-specific VO_2peak_ (65%).

There were no differences in ventilatory responses (i.e., VE, VO_2_, VCO_2_, VE · VO_2_^-1^, VE · VCO_2_^-1^, or SaO_2_) between groups at 45% and 65% of altitude-specific VO_2peak_ at SL or in HYP. As expected, the exercise VO_2_, VCO_2_, and SaO_2_ were lower (*P* < 0.05), and VE was similar in HYP compared to SL within groups at the same relative work rates. The VE · VO_2_^-1^ and VE · VCO_2_^-1^, therefore, were both increased (*P* < 0.05) in HYP compared to SL at 45% and 65% of altitude-specific VO_2peak_.

There were no differences in any of the cardiovascular or perceptual responses (i.e., HR, CO, MAP, and RPE) between groups at 45% and 65% of altitude-specific VO_2peak_ at SL or in HYP. Within groups, there were also no changes in any of these variables from SL to HYP at either of the relative work rates. Also, the percent change in CO from SL to HYP within groups did not differ between HH and NH. Individuals with a lower exercise SaO_2_ during steady-state exercise were related to individuals with a higher CO at 45% (*r* = -0.45, *P* = 0.04) and 65% (*r* = -0.39, *P* = 0.06) altitude-specific VO_2peak_ in HYP. The percent change in PV from pre- to post-exercise was not different in the HH and NH groups at SL (-9.0 ± 3.4 vs. -11.9 ± 6.3) or HYP (-9.1 ± 6.0 vs. -8.1 ± 4.8), respectively.

### Time trial performance data

**Figure 2:**
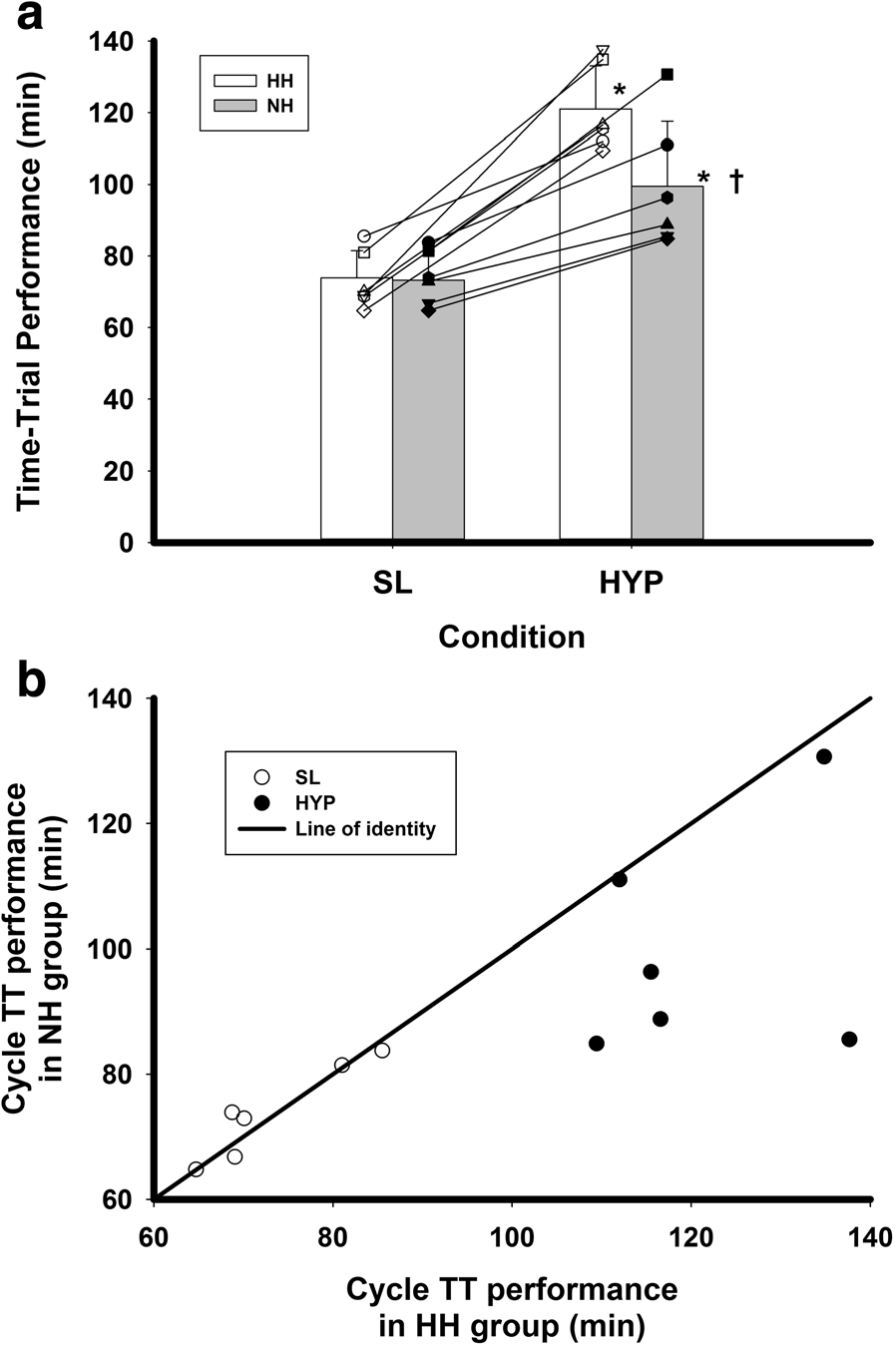
**Time trial performance data. (a)** Cycle time trial performance at sea level (SL) and hypoxia (HYP) in a group of men exposed to either hypobaric hypoxia (HH) or normobaric hypoxia (NH). Each man exposed to HH demonstrated a greater decrement in TT performance in hypoxia (HYP) than each man exposed to NH at an ambient PO_2_ equivalent to 4,300 m in each condition. **(b)** For each matched pair, their sea level (SL) TT performance fell closely on the line of identity, but their performance in HH fell below their matched pair in NH on the line of identity. *P < 0.05 from SL; ^†^*P* < 0.05 between groups.

Both the HH and NH groups exhibited similar TT performance at SL. Not only was the mean TT performance similar between groups, but also individuals in the HH and NH groups were closely matched on their TT performance at SL (Figure 2a). The TT performance, however, was longer (*P* < 0.05) in HH compared to NH (Figure 2a). The percent decrement in TT performance from SL to HH (65.1 ± 23.6%) was also much greater (*P* < 0.05) than the decrement from SL to NH (35.5 ± 13.7%). Moreover, for each pair of men matched on physical performance and demographic characteristics, the individual exposed to HH had a greater decrement in TT performance than the one exposed to NH (Figure 2b). The mean power outputs maintained for the HH and NH group, respectively, during the first (163 ± 19 W vs. 164 ± 16 W), second (166 ± 21 W vs. 164 ± 24 W), third (163 ± 18 W vs. 161 ± 17 W), and fourth (164 ± 17 W vs. 175 ± 16 W) quarters of the TT conducted at SL were not different. The power outputs maintained in the HH and NH group, respectively, during the first (104 ± 17 W vs. 129 ± 22 W), second (100 ± 8 W vs. 123 ± 14 W), third (96 ± 10 W vs. 117 ± 21 W), and fourth (102 ± 9 W vs. 127 ± 28 W) quarters of the TT conducted in HYP tended (*P* = 0.08) to be approximately 20 W lower in the HH group compared to the NH group.

During the TT, the mean SaO_2_ was similar in the HH and NH groups at SL (97 ± 2% vs. 97 ± 1%) and in HYP (74 ± 5% vs. 77 ± 3%; *P* = 0.07), respectively. The HR during the TT was also similar in the HH and NH groups at SL (168 ± 13 bpm vs. 165 ± 12 bpm) and in HYP (150 ± 9 bpm vs.158 ± 13 bpm), respectively. The RPE during the TT was similar in the HH and NH groups at SL (14 ± 3 vs. 15 ± 2) and in HYP (14 ± 2 vs. 15 ± 1), respectively.

## Discussion

The major finding from this study was that cycle TT performance was impaired to a greater degree in HH compared to NH which supports the first part of our hypothesis. As such, exposure to NH may not induce the same hypoxic stimulus and training benefit as exposure to HH. The two groups were closely matched at sea level on physical performance and demographic characteristics which makes the observed TT performance differences between HH and NH more convincing. Moreover, the fact that each individual exposed to HH demonstrated a larger decrement in TT performance compared to their fitness and age-matched pair exposed to NH demonstrates that this response was consistent across a wide range of individuals. Our findings do not support the second part of our hypothesis that increased ventilation and improved O_2_ delivery in NH compared to HH were responsible for the differences in TT performance. In fact, nothing we measured was able to identify the reasons for the TT performance differences between HH and NH. This result suggests the need for additional physiologic and metabolic measures (e.g., cerebrovascular regulation and oxygenation, nitric oxide bioavailability, subclinical pulmonary edema) to determine the reason for such large differences in TT performance [19,34].

Our ventilatory data during rest, steady-state exercise at 45% and 65% of altitude-specific VO_2peak_, and the cycle TT do not indicate clear differences between groups. The resting PETCO_2_ was the same in HH and NH in the present study, which supports the findings of some [16,20] but not others [17,19,21]. Differences between studies may be related to the use of partially acclimatized subjects in studies reporting differences [17,21]. Given that resting SaO_2_ was not different between HH and NH in any of the reported studies, the functional significance of a slightly lower resting PETCO_2_ in NH is not conclusive. During steady-state exercise in the present study, PETCO_2_ was not measured but the ventilatory equivalents for O_2_ and CO_2_ were not different between groups at either of the two work rates. In addition, the SaO_2_ was not different between groups during steady-state exercise at either of the work rates or during the cycle TT. Our results confirm and extend the previously reported lack of differences in resting SaO_2_ in the two environments [17-19] to no differences during steady-state exercise at 45% and 65% of altitude-specific VO_2peak_ or during a cycle TT. It seems clear that if differences do exist in resting or exercise ventilation, they are subtle and do not affect the end product of pulmonary gas exchange.

Our cardiac and perceptual response data during rest, steady-state exercise at 45% and 65% altitude-specific VO_2peak_, and the cycle TT also do not indicate clear differences between groups. Others have also reported no differences in either resting HR, CO, or MAP between the two environments [18-22], while one study reported a higher resting HR in HH compared to NH [16]. No studies have compared RPE between the two environments. Our results are consistent with a previously reported lack of differences in resting cardiac responses to the two environments [23] and extend these findings to no differences during steady-state exercise or during a cycle TT. The cycle TT performance differences observed in the present study, therefore, cannot be attributed to differences in cardiac output or oxygen delivery between HH and NH.

Given that volunteers were able to self-select the power output at the beginning and during the cycle TT, we analyzed pacing strategies between groups during different quarters of the TT. The volunteers in the HH group tended (*P* = 0.08) to select an approximately 20-W lower power output compared to the NH group right from the beginning of the TT. Even though the difference was not statistically significant, it suggests that perceptual clues were influencing their self-selected power output prior to even starting TT exercise. The HH group also perceived a lower power output as being equally hard despite a tendency for a lower HR compared to the NH group. This finding suggests a subjective feeling that exercise is more difficult in HH compared to NH.

On an individual basis, a lower SaO_2_ during steady-state exercise was associated with a higher CO in both the HH and NH groups in this study. This finding suggests that a greater level of hypoxemia will result in compensatory vasodilatation to match O_2_ supply to demand. This finding has been previously reported [35]. However, the response was not dependent on the environment and as such cannot account for the large differences in TT performance between groups. A recent study [36] reported that hypocapnia may decrease cerebral blood flow and thus cerebral oxygenation which could become a limiting factor in exercise performance if SaO_2_ falls below 82% [37]. In the present study, PaCO_2_ was not measured, but one study [20] reported a lower PaCO_2_ in HH compared to NH despite a similar PETCO_2_. If this occurred in the present study, our SaO_2_ values during exercise were well below the 82% cutoff value and a greater degree of hypocapnia in HH may have induced a greater decrement in TT performance.

Although greater fluid retention due to less diuresis has been reported in HH compared to NH [38,39], our data do not suggest any differences in fluid balance between the two environments. The change in PV from SL to HYP was similar in both HH and NH, and the change in PV during exercise was also similar in both environments. Previous research demonstrated compelling radiographic evidence of pulmonary edema in athletes exercising in HH [40], while other studies found no lung water accumulation in humans exercising in NH [41,42]. Differences in fluid circulation and the trans alveolar-capillary membrane flux [43] may induce greater pulmonary vasoconstriction in HH and decrease O_2_ diffusion via a decreased pressure gradient [13]. This decrease in O_2_ diffusion could potentially limit exercise performance and explain the greater TT impairment in HH compared to NH. In addition, nitric oxide bioavailability has been reported to be lower in HH compared to NH [19] which could impair O_2_ unloading at the tissue and negatively impact TT performance in HH. Nitrate supplementation has been shown to improve exercise tolerance in hypoxia and reduce muscle metabolic perturbation due to improved O_2_ delivery [44]. Further research is needed to elucidate potential physiologic mechanisms responsible for the large observed differences in TT performance between HH and NH.

Limitations of this study should be acknowledged. First, although our sample size was adequate to detect differences in cycle TT performance, borderline differences in SaO_2_, HR, and power outputs during the TT may have become significant if a larger sample size was utilized. A recent article justifying small-*n* research sheds light on the importance of analyzing trends, and the current trend (despite a lack of statistical significance) should not be disregarded [45]. Second, the data were collected within the first 4 h of exposure. Thus, our physiologic findings should not be extended beyond that time point. Third, the ambient PO_2_ was kept the same, but PIO_2_ can differ between groups based on the differences in water vapor calculations [34]. In this study, the calculated PIO_2_ was slightly lower in HH (83.4 mmHg) compared to NH (87.4 mmHg). However, the resting SaO_2_ was similar in both groups, indicating that the same level of hypoxemia, regardless of PIO_2_, was induced in both environments. Fourth, a 26% decrement in VO_2peak_ was assumed in both HH and NH. There is individual variability in this decrement at altitude [46], and if VO_2peak_ was decremented to a larger degree in HH than NH, the volunteers may have started their TT in a more fatigued state in HH. The RPE, however, did not differ between groups during the submaximal work rates or during the time trial. Last, independent groups of volunteers were utilized. A cross-over design was not utilized due to potential carry-over effect of a previous exposure to hypoxia on performance. The volunteers, however, were closely matched on fitness levels and demographic characteristics, which make the results more convincing.

The practical implications of this study are important for athletes, mountaineers, military personnel, and others employing NH to induce health and performance benefits at terrestrial altitude. Although the physiologic responses appear to be similar between the two environments, it is clear that TT performance is impacted to a greater degree in HH. If the objective of training programs is to improve performance at terrestrial altitude, HH appears to be a more stressful stimulus and would likely induce greater performance adaptations. The specificity of training principle as well as a recent review article citing no improvements in endurance performance, measured in hypobaric hypoxia, following intermittent exposures to NH but great improvements with intermittent exposures to HH supports this argument [8].

## Conclusions

In conclusion, this study found a greater decrement in cycle TT performance in HH compared to NH. Clearly, HH appears to be a more stressful stimulus, and NH should not be utilized as a substitute for HH when endurance performance is the main outcome variable of interest. Ventilatory, cardiovascular, and hematologic responses did not differ during rest, steady-state exercise at the same relative or absolute work rates, or a cycle TT performance test between environments. Further research is needed to elucidate potential physiologic mechanisms responsible for the observed differences in TT performance between HH and NH.

## Abbreviations

CO: Cardiac output; DBP: Diastolic blood pressure; HH: Hypobaric hypoxia; HR: Heart rate; HYP: Hypoxia; MAP: Mean arterial pressure; NH: Normobaric hypoxia; PETCO2: Partial pressure of end-tidal carbon dioxide; PETO2: Partial pressure of end tidal oxygen; PIO2: Inspired PO_2_; PO2: Ambient PO_2_; PV: Plasma volume; RPE: Rating of perceived exertion; SaO2: Arterial oxygen saturation; SBP: Systolic blood pressure; SL: Sea level; TT: Time trial; VE: Minute ventilation; VE · VCO2-1: Ventilatory equivalent for carbon dioxide; VE · VO2-1: Ventilatory equivalent for oxygen; VCO2: Carbon dioxide production; VO2: Oxygen consumption; VO2peak: Peak oxygen consumption.

## Competing interests

The authors declare that they have no competing interests.

## Authors' contribution

BAB, CSF, and SRM conceived and designed the study, collected and analyzed the data, and wrote the manuscript. JES and SPA collected and analyzed the data. All authors read and approved the final manuscript.
